# Putting communities at the forefront of community-led monitoring in Zimbabwe

**DOI:** 10.3389/fpubh.2023.1320944

**Published:** 2024-01-08

**Authors:** Tatenda Makoni, Bernard Madzima, Tafadzwa Dzinamarira, Enos Moyo, Amon Mpofu, Innocent Chingombe, Munyaradzi Mapingure, Godfrey Musuka

**Affiliations:** ^1^Zimbabwe National Network of People Living with HIV, Harare, Zimbabwe; ^2^National AIDS Council, Harare, Zimbabwe; ^3^School of Health Systems and Public Health, University of Pretoria, Pretoria, South Africa; ^4^School of Nursing and Public Health, University of KwaZulu Natal, Durban, South Africa; ^5^Innovative Development and Public Health, Harare, Zimbabwe; ^6^International Initiative for Impact Evaluation, Harare, Zimbabwe

**Keywords:** HIV, community-led monitoring, Zimbabwe, monitoring, evaluation

## Introduction

The purpose of this opinion manuscript is to outline the role of community-led organizations in the fight against HIV including the monitoring of HIV-related health services in Zimbabwe. This is in line with the theme of the 2023 World AIDS Day of recognizing and celebrating the achievements of communities. Additionally, the World AIDS Day theme advocates for more resources to enable community-led organizations to unleash their full potential and leadership, including more effective community-led monitoring of the provision of HIV-health services to contribute to the end of AIDS by 2030.

In 2014, The Joint United Nations Programme on HIV/AIDS (UNAIDS) launched the 95-95-95 targets. The aim was to diagnose 95% of all HIV-positive individuals, provide antiretroviral therapy (ART) for 95% of those diagnosed, and achieve viral suppression for 95% of those treated by 2030. Having achieved the 95-95-95 UNAIDS targets with time to spare, Zimbabwe is on the trajectory to ending HIV/AIDS and, in turn, coming closer to achieving its health-related Sustainable Development Goals (1). Zimbabwe's success is attributable to factors including, strong political will, and employment of robust and innovative community-level HIV testing, treatment, and care service provision for both the general population and key populations. For example, for key populations, the country has used granular data and HIV risk profiling to ensure that interventions reach those at great risk of HIV acquisition. The country's approach highlights the necessity of localized targeted interventions that address sociodemographic constraints and geographic heterogeneity in HIV service provision.

In our view, HIV community-led monitoring (CLM), an accountability mechanism for the HIV response at different levels, led and implemented by local community-led organizations (CLOs) of people living with HIV (PLHIV), networks of key populations, other affected groups or other community entities, will be a crucial strategy to promote sustainability (2). In Zimbabwe, Community lead Monitoring has proved to be an effective mechanism for organized and systematic advocacy for the health and rights of key populations (3). Engaging peers in service delivery is an effective strategy to reduce stigma among key populations. Obtaining feedback from recipients of care can inform healthcare workers on how to improve the quality of service delivery and address issues such as stigma, that cause low uptake of services and poor health outcomes.

## Current gaps in CLM in Zimbabwe

Despite the country having registered some progress in the implementation of CLM, the following gaps are still apparent and require attention to enhance the quality and utility of CLM-generated data;

*Limited capacity of CLOs:* Many CLOs in Zimbabwe lack the resources to implement CLM effectively as well as some of the necessary technical skills, particularly in areas including data management, and reporting.

*Sustainability challenges:* CLM is often funded by external donors, which raises concerns about its sustainability once donor funding dries up. One of the contributing factors to this gap is a general lack of advocacy efforts by the CLOs, that target the private sector players to be actively involved in supporting health interventions that affect communities that support their businesses. There is a need to develop innovative financing mechanisms to ensure the long-term sustainability of CLM in Zimbabwe.

*Limited engagement with government:* Government engagement in CLM is still relatively limited in Zimbabwe, despite being the key stakeholder in the HIV response. The limited involvement of the government in the implementation of CLM can result in the lack of policies and structures to support the programme, a problem that can lead to its premature collapse. There is a need to strengthen engagement between CLOs and the government to ensure that CLM findings are used to inform policy and programming.

*Data quality challenges:* The quality of data collected through CLM in Zimbabwe still leaves a lot to be desired, with aspects of data quality including completeness, timeliness and consistency among others, affected. This is due to factors, including, the lack of training on data collection and analysis tools, and the lack of standard methodologies among different CLOs. There is a need to develop standardized CLM tools and methodologies to improve the quality of data collected.

## Recommendations for strengthening CLM in Zimbabwe

To strengthen CLM in Zimbabwe and ensure that it becomes more effective, the following recommendations are put forward;

1. For some of the key gaps outlined in the section above to be addressed, there is a need to provide training for community organizations to strengthen their activities supporting PLHIV. Policymakers in Zimbabwe must support these organizations with funding and training opportunities to ensure that their work achieves the desired impact. Underfunding of community-led initiatives will result in their activities achieving limited impact.
2. The government of Zimbabwe must continuously consider how to make community initiatives more sustainable by increasing the allocation of domestic resources raised by the National AIDS Council from the National AIDS Levy Trust Fund (NALTF). The NALTF was established by the Zimbabwe legislature in 1999, the AIDS Levy entails a 3% income tax for individuals and a 3% tax on profits of employers and trusts (which excluded the mining industry until 2015). It is managed by the parastatal NAC through a decentralized structure of AIDS Action Committees. The NALTF amongst others contributes 15% of the financial resources for the procurement of the national ARV requirements.
3. Strengthen monitoring of treatment literacy: Effective community-level HIV treatment literacy is essential to support Zimbabwe's robust HIV response. CLM must strengthen its monitoring of treatment literacy, especially when the general population regards HIV/AIDS under control. Treatment literacy means that people, individually and in communities, understand what HIV drugs are, why they are needed, and what they can and cannot do. As the HIV-positive cohort in Zimbabwe ages and non-communicable diseases (NCDs) become more prevalent, treatment literacy must address this dimension.
4. CLM needs to provide greater granularity so that the needs of different types of health facilities, urban vs. rural, state-owned vs. NGO or religious-owned health facilities, and other strata, are accurately articulated during routine rounds of CLM monitoring. This will allow for the rapid identification and address of specific shortcomings in service provision.
5. Community-based monitoring (CBM) refers to service users assessing the effectiveness, quality, accessibility, and impact of health programs and services they receive. Conversely, CLM is a specific type of CBM that is led and implemented by CLOs of PLHIV, networks of key populations, other affected groups, or other community entities. CLM is important because it puts communities at the forefront of HIV health service delivery monitoring. CLOs have a unique understanding of the needs and challenges faced by their communities, and they are well-positioned to identify and address gaps in service provision. Funders of HIV programs should ensure that resources continue to flow to CLOs, as they play a vital role in CLM. CLOs may not have the administrative strength and depth of international organizations and large national NGOs. However, this should not be held against them, as they are closest to the individuals who receive HIV-related health services at facilities dotted around the country. Any gaps in program quality and service delivery models that do not meet the needs and expectations of PLHIV threaten individual outcomes and public health goals. CLM is an essential tool for identifying and addressing these gaps.
6. Differentiated service delivery (DSD) is a client-centred approach that simplifies and adapts HIV services across the cascade to reflect the preferences, expectations, and needs of people living with and vulnerable to HIV while reducing unnecessary burdens on the health system (4). DSD provides an opportunity for the HIV and Universal Health Coverage agendas and is increasingly evolving to a more client-centred vision of chronic care services, which includes reducing the number of facility visits for stable HIV clients (5). CLM implementation in Zimbabwe has tended to focus on health facilities, leaving out those individuals on DSD who, on average, visit the facility every 6 months to get their refills. Service delivery concerns of individuals on DSD are less likely to be captured by CLM as these individuals rarely visit health facilities and are, therefore, less likely to participate in CLM data collection surveys.

CLM needs to identify innovative approaches so that those stable clients on DSD approaches can also join in CLM data collection surveys. Some possible approaches include:

- Using mobile phone surveys: Mobile phone surveys can be a convenient and effective way to collect data from individuals on DSD, especially those who live in rural areas or have difficulty travelling to health facilities.
- Partnering with community-based organizations (CBOs): CBOs can be vital in identifying and engaging individuals on DSD in CLM data collection surveys. CBOs have a strong presence in communities and can help to build trust with individuals on DSD.
- Using peer educators: Peer educators can educate individuals on DSD about the importance of CLM and assist them in participating in CLM data collection surveys.

It is important to note that any approach used to collect data from individuals on DSD should respect their time and needs. It is also essential to ensure that the data collected is confidential and secure.

7. CLM activities have also been introduced not only for HIV but also for TB and Malaria programs. There is a need for discussions about reducing redundancy between these CLM programs and ensuring that value for money is achieved. One way to minimize redundancy is coordinating CLM activities across different disease programs. This could be done by developing standard CLM tools and methodologies and sharing data and lessons learned. Another way to reduce redundancy is to focus on CLM activities that significantly impact health outcomes. For example, CLM could be used to monitor the quality of care provided for key populations, such as sex workers, and people who use drugs. Finally, it is essential to ensure that CLM is implemented cost-effectively. This could be done using innovative data collection methods like mobile phone surveys and partnering with CBOs.
8. For CLM to have the desired impact and leverage and for it to be able to result in the rapid and effective resolution of HIV service delivery and quality bottlenecks at the health facility level, networks of PLHIV need to increase their engagement with other key players at the community level, such as traditional leaders. Traditional leaders have a different type of agency and better access to the levers of government to cause positive change in health service delivery, which groupings of PLHIV may not have. Traditional leaders must be well-informed about HIV health service delivery challenges in their communities to contribute to advocacy activities. Additionally, traditional leaders can apply resources they receive from the central government to address health service delivery bottlenecks, such as abolishing user fees at the local level or crowdfunding to maintain or install health facility-level infrastructure such as boreholes. In Zimbabwe, traditional leaders make up approximately a quarter of the membership in the Senate and can therefore be important advocates for health service delivery matters for PLHIV. They can assist PLHIV networks in advocating to the central government to address local health service provision bottlenecks.

We also propose some specific approaches for how CLM networks and traditional leaders can work together to improve HIV service delivery:

- Conduct joint assessments of HIV service delivery: CLM networks and traditional leaders can work together to conduct joint assessments of HIV service delivery in their communities. This will help to identify common challenges and areas for improvement.
- Develop and implement joint advocacy plans: CLM networks and traditional leaders can work together to develop and implement joint advocacy plans to address the challenges identified in the joint assessments. These advocacy plans could target local government officials, policymakers, and other stakeholders.
- Mobilize communities to support HIV service delivery: Traditional leaders can use their influence to mobilize communities to support HIV service delivery. This could involve encouraging community members to participate in CLM activities, to volunteer their time and skills at health facilities, and to donate resources to support HIV programs.

## Conclusion

Zimbabwe has achieved the 95-95-95 UNAIDS targets, a significant milestone in the fight against HIV/AIDS. However, it is essential to remain vigilant and invest in strategies to ensure that the gains made are not lost. Too often, decision-makers treat communities as problems to be managed, rather than as leaders to be recognized and supported. The HIV response is hurt when community leadership—the greatest power for progress—is unacknowledged, undersupported, underresourced, underremunerated, and in some places even under attack. Additionally, it has been a long-standing principle of the HIV response for people living with or affected by HIV to have a place at the decision-making table. CLM is a critical strategy for promoting sustainability in the HIV response. It puts PLHIV at the forefront of monitoring and advocacy efforts. This helps to ensure that the needs of PLHIV are heard and that services meet their needs. PLHIV-led organizations are responsible for implementing CLM activities in their communities. In addition to PLHIV-led organizations, other community players, such as traditional leaders, also play a role in CLM implementation, with Traditional leaders offering a unique understanding of their communities and helping to mobilize support for its activities. As Zimbabwe strives to achieve sustainability and eliminate HIV/AIDS before 2030, it is essential to re-double efforts to ensure that CLM is well-resourced. PLHIV-led organizations and other community players must be supported to implement effective CLM activities. To ensure the sustainability of Zimbabwe's HIV response, there is a need for community initiatives led by key organizations such as ZNNP+ to be funded using domestic financial resources (taxes and levies) collected by the NALTF. By making more sustainable funding available to community initiatives, the country will be more likely to maintain the 95-95-95 target achievement and ultimately work toward ending the HIV/AIDS epidemic as a public health threat by 2030, Finally, in line with World AIDS Day 2023 theme, communities' leadership roles need to be made core in all HIV plans and programmes. They need to be fully and reliably funded. Barriers to communities' leadership roles need to be removed (6).

## Author contributions

TM: Writing – original draft. BM: Writing – review & editing. TD: Writing – review & editing. EM: Writing – review & editing. AM: Writing – review & editing. IC: Writing – review & editing. MM: Writing – review & editing. GM: Conceptualization, Writing – original draft.
